# Respiratory Infections Are More Common Than Healthcare Records Indicate: Results From an Anonymous Survey

**DOI:** 10.1093/milmed/usac016

**Published:** 2022-02-03

**Authors:** Stephanie A Richard, Patrick J Danaher, Brian White, Katrin Mende, Rhonda E Colombo, Timothy H Burgess, Christian L Coles

**Affiliations:** Infectious Disease Clinical Research Program, Department of Preventive Medicine and Biostatistics, Uniformed Services University of the Health Sciences, Bethesda, MD 20814, USA; The Henry M. Jackson Foundation for the Advancement of Military Medicine, Inc., Bethesda, MD 20817, USA; Air Force Medical Readiness Agency, Falls Church, VA 22042, USA; Brooke Army Medical Center, JBSA Fort Sam Houston, TX 78234, USA; Infectious Disease Clinical Research Program, Department of Preventive Medicine and Biostatistics, Uniformed Services University of the Health Sciences, Bethesda, MD 20814, USA; The Henry M. Jackson Foundation for the Advancement of Military Medicine, Inc., Bethesda, MD 20817, USA; Brooke Army Medical Center, JBSA Fort Sam Houston, TX 78234, USA; Infectious Disease Clinical Research Program, Department of Preventive Medicine and Biostatistics, Uniformed Services University of the Health Sciences, Bethesda, MD 20814, USA; The Henry M. Jackson Foundation for the Advancement of Military Medicine, Inc., Bethesda, MD 20817, USA; Madigan Army Medical Center, Joint Base Lewis-McChord, WA 98431, USA; Infectious Disease Clinical Research Program, Department of Preventive Medicine and Biostatistics, Uniformed Services University of the Health Sciences, Bethesda, MD 20814, USA; Infectious Disease Clinical Research Program, Department of Preventive Medicine and Biostatistics, Uniformed Services University of the Health Sciences, Bethesda, MD 20814, USA; The Henry M. Jackson Foundation for the Advancement of Military Medicine, Inc., Bethesda, MD 20817, USA

## Abstract

**Introduction:**

Influenza-like illnesses (ILIs) are common in military populations and can impair mission-readiness, particularly in the current severe acute respiratory syndrome coronavirus 2 pandemic; therefore, it is important to identify potential risk factors for infection and better understand the burden of infection.

**Materials and Methods:**

A survey was administered to military medical trainees living in a congregated setting on JBSA Fort Sam Houston, Texas, from January 2017 to February 2019. The survey included questions about ILI experience and potential ILI risk factors.

**Results:**

2,121 individuals completed the survey. Respondents had a median age of 21 years, 46% were female, 32.6% were Air Force, 33.6% were Army, and 33.8% were Navy/Marines. Among the 815 (38%) who reported an ILI during training, 40% sought health care. The primary reasons for seeking healthcare included illness severity, concern about transmission, and accessibility of healthcare. Over half (54%) of the trainees who reported an ILI said the ILI had an impact on their performance, including reduced study time, missed physical training, and missed class. Multivariate model results indicate that women and younger trainees (<30 years) were more likely to report having had an ILI (women: OR 1.58, (95% CI 1.30, 1.92); age <30 years: OR 1.58, (1.06, 2.36)). In a subset analysis, those who reported washing their hands 10+ times per day were less likely to report an ILI (OR 0.61 (0.42, 0.89)).

**Conclusions:**

ILIs are likely to be more common during training than healthcare records indicate and may result in decreased training effectiveness. Increasing access to handwashing facilities and education about the importance of handwashing to prevent the spread of disease will likely reduce the ILI burden in this population.

## INTRODUCTION

Lower respiratory infections remain one of the leading causes of morbidity and mortality worldwide,^1^ particularly during the ongoing severe acute respiratory syndrome coronavirus 2 (SARS-CoV-2) pandemic. Respiratory infections are a particular problem in the U.S. military where, despite high (>90%) influenza and adenovirus vaccination rates, respiratory infections are responsible for 25% to 30% of infectious disease hospitalizations^2^ and can impair mission readiness.^3^ U.S. military members have high burdens of respiratory infections as a result of frequent travel, close living situations, and physically strenuous trainings, which can affect transmission of and susceptibility to respiratory infections.^4,5^ While vaccination for influenza and adenovirus provides some protection,^6,7^ influenza vaccine effectiveness in the U.S. military in 2017-2018 (18%^8^) has been demonstrated to be lower than that in the general U.S. general population (18-49 years of age) (33%^9^) and the reasons for this disparity are unclear.

Some U.S. military locations provide advanced training to active duty personnel. For example, there are approximately 5,500 trainees at the Medical Education and Training Campus (METC) on JBSA Ft. Sam Houston at any given time, all of whom live in college-style dormitories on base and attend classes that range from 4 to 52 weeks in length, depending on their specialties. The transience of the population combined with the close living and sleeping facilities during training provide an ideal environment for respiratory infection transmission.^3^ Unlike university settings with a common starting date in the fall during which influenza-like illness (ILI) outbreaks can occur,^10^ individuals at these training centers are arriving and leaving year-round. Clinic visits can be used to assess the incidence of ILI in this population, but there is likely an additional burden of non-medically attended ILI among trainees due to milder symptoms and/or fear of missing training. The primary goal of this research is to describe the burden of ILI in a military trainee population and to identify sociodemographic and lifestyle factors associated with self-reported ILI, in order to develop preventive measures and enhance mission readiness in the U.S. military.

## METHODS

Anonymous surveys were administered in a convenience sample of active duty personnel receiving advanced medical training at METC at JBSA Ft. Sam Houston (San Antonio, TX) from January 2017 through March 2019. The anonymous surveys were administered to a representative sample of pending graduates at the same time as the regular end-of-course evaluations were conducted. Two versions of the survey were administered during the study. Both versions of the survey included questions about sociodemographic characteristics (age, sex, race/ethnicity, and military status and affiliation), as well as some basic questions about ILI symptoms (defined as fever/feverish feeling plus cough and/or sore throat) and potential risk factors (presence of symptoms, healthcare-seeking behaviors, hygiene behaviors, etc.). Version 2 of the survey, implemented in January 2018, included additional questions about the rationale behind seeking healthcare (or not seeking healthcare) for their ILI, the rationale behind not using hand sanitizer or washing hands, and the impact the ILI had on the performance of the respondent. In addition, version 2 of the survey added a new category capturing the number of times the participant reported washing their hands per day.

Responses were summarized as mean values for continuous measures and number (%) for binary and categorical variables. Trainees who reported ILI symptoms were compared with those who did not report ILI symptoms using Kruskal-Wallis rank sum test for continuous variables and Pearson’s Chi-squared tests for categorical variables (Table I). We ran multivariable logistic regression models to assess those factors that were associated with ILI among the trainees, controlling for length of training, calendar month of survey (distribution in Supplementary Table S1), and influenza season (2016/17, 2017/18, or 2018/19). In addition, a sub-analysis was run only on those who filled out version 2 of the form, based on greater granularity of the handwashing questions. A multivariable logistic regression model was also run to identify those factors associated with healthcare-seeking behavior, among those who reported having had an ILI, also controlling for length of training, month of survey, and influenza season (2016/17, 2017/18, or 2018/19). For all logistic models, we considered univariable relationships as well as scientific rationale when choosing which variables to test in the model and assessed the models’ fit using likelihood ratio tests. Analyses were performed using R v.4.0.4.^11^

**TABLE I.** Characteristics of Population in Respiratory Infection Study at Fort Sam Houston, by Influenza-like Illness (ILI) Status

	Did not report an ILI (*N* = 1,306)	Reported an ILI (*N* = 815)	Total (*N* = 2,121)	*P* value
Median age, years (Q1, Q3)	21 (19, 24)	20 (19.0, 23)	21 (19, 24.0)	<.01^1^
<30 years old	1,188 (91.0%)	778 (95.5%)	1,966 (92.7%)	<.01^2^
Female	568 (43.5%)	409 (50.2%)	977 (46.1%)	<.01^2^
Race/ethnicity				<.01^2^
Asian	104 (8.0%)	61 (7.5%)	165 (7.8%)	
Black	286 (21.9%)	119 (14.6%)	405 (19.1%)	
Hispanic	290 (22.2%)	164 (20.1%)	454 (21.4%)	
Multiple/other race	119 (9.1%)	75 (9.2%)	194 (9.1%)	
White	507 (38.8%)	396 (48.6%)	903 (42.6%)	
Military status				.99^2^
Active duty	960 (73.5%)	600 (73.6%)	1,560 (73.6%)	
Reservist/National Guard	319 (24.4%)	199 (24.4%)	518 (24.4%)	
Other or missing	27 (2.1%)	16 (2.0%)	43 (2.0%)	
Branch				<.01^2^
Air Force	492 (37.7%)	199 (24.4%)	691 (32.6%)	
Army	427 (32.7%)	286 (35.1%)	713 (33.6%)	
Navy/Marines	387 (29.6%)	330 (40.5%)	717 (33.8%)	
Healthcare sought for ILI symptoms	–	328 (40.2%)	328 (40.2%)	–
Median length of training, days (Q1, Q3)	77 (50, 95)	91 (51, 105)	79 (50, 98)	<.01^1^
Season				<.01^2^
2016/2017	467 (35.8%)	498 (61.1%)	965 (45.5%)	
2017/2018	695 (53.2%)	266 (32.6%)	961 (45.3%)	
2018/2019	144 (11.0%)	51 (6.3%)	195 (9.2%)	
Active 3+ times per week	1,119 (85.7%)	731 (89.7%)	1,850 (87.2%)	<.01^2^
Smoking status				.55^2^
Current	75 (5.7%)	44 (5.4%)	119 (5.6%)	
Former	158 (12.1%)	115 (14.1%)	273 (12.9%)	
Missing	9 (0.7%)	7 (0.9%)	16 (0.8%)	
Never	1,064 (81.5%)	649 (79.6%)	1,713 (80.8%)	
Washes hands 4+ times per day	1,169 (89.5%)	709 (87.0%)	1,878 (88.5%)	.08^2^
Does not wash hands after coughing or sneezing	346 (26.5%)	278 (34.1%)	624 (29.4%)	<.01^2^
Subset with version 2 of survey and reported that they do not wash hands after coughing or sneezing	122/773 (15.8%)	58/288 (20.1%)	180/1,061 (17.0%)	
If the participants responded that they do not wash hands after coughing or sneezing, responses to “If No, why don’t you usually wash your hands or use hand sanitizer?” (Check all that apply)				
Did not think about it	62 (50.8%)	32 (55.2%)	94 (52.2%)	.58^2^
Handwashing facilities/sanitizer not available	27 (22.1%)	12 (20.7%)	39 (21.7%)	.83^2^
Sneezed or coughed in elbow, no need to wash	26 (21.3%)	14 (24.1%)	40 (22.2%)	.67^2^
Takes too much time	14 (11.5%)	6 (10.3%)	20 (11.1%)	.82^2^
Other	3 (2.5%)	0 (0.0%)	3 (1.7%)	.23^2^

1 1. Kruskal-Wallis rank sum test

2 2. Pearson’s Chi-squared test

## PATIENT CONSENT STATEMENT

This study was conducted in accordance with the Declaration of Helsinki and good clinical practice guidelines and written informed consent was provided before administration of survey by all study participants. This study was approved by the Uniformed Services University Institutional Review Board (IDCRP-103).

## RESULTS

A total of 2,121/2,417 (88%) of the surveys included age, sex, ILI history, and length of training and were considered in the analysis, among whom 1,153 trainees completed the extended survey (version 2 of the survey, implemented in January 2018). The trainees were, on average, in their early 20s, 46% female, and represented the Air Force, Army, and Navy/Marines (Table I). Smoking was uncommon (6%); 87% of the total population reported themselves to be physically active three or more times per week. Training duration was approximately 3 months, on average, but ranged from 3 weeks to over 1 year in some cases.

Many of those surveyed reported washing their hands at least four times per day (89%), although fewer reported washing their hands after sneezing or coughing (71%). When a subset of trainees was asked why they did not wash their hands after sneezing or coughing, 52% reported that they did not think about it, 22% reported that facilities for handwashing or sanitizing were not available, and 22% sneezed or coughed into their elbow, therefore obviating the need to wash their hands (Table I).

Thirty-eight percent of those surveyed (815/2,121) reported having experienced an ILI during their training, and 40% of those who had an ILI (328/815) reported seeking healthcare for those symptoms. Among the extended survey trainees, the primary reasons for seeking healthcare included the severity of the illness (59%), concern about spreading the illness (49%), and the accessibility of healthcare (40%). Those who did not seek healthcare reported concerns about missing class (84%), not having a severe illness (62%), and being busy (43%) as reasons for not seeking healthcare (Table II). Over half of the extended survey trainees reported that their ILI had an impact on their performance, 53% of those missed class, 77% of those reported decreased study time, 68% missed physical training, and 79% decreased leisure activities. No statistically significant difference was observed for race, activity level, handwashing, or travel history. Only 15% of participants reported not taking any medication to treat their ILI.

**TABLE II.** Responses to Questions about Influenza-like Illnesses (ILI) Asked on Version 2 of the Survey, among Those Who Reported an ILI

	*N* = 289
Healthcare sought for ILI symptoms	143 (49.5%)
Why did you seek healthcare? (choose as many as apply)	
The illness was severe	85 (59.4%)
Did not want to spread illness to others	70 (49.0%)
Healthcare was easily accessible	57 (39.9%)
Healthcare was affordable	29 (20.3%)
Pressure from others to seek healthcare	25 (17.5%)
How many days did you wait to see a provider after you started to feel sick?	
0 day (on the same day)	15 (10.6%)
1-3 days	63 (44.4%)
4-7 days	25 (17.6%)
More than 7 days	39 (27.5%)
*N* Missing	1
Healthcare not sought for ILI symptoms	146 (50.5%)
If the participant did not seek healthcare, what were the reasons for not seeking healthcare? (choose as many as apply)	
Did not want to miss class	123 (84.2%)
The illness was not that severe	90 (61.6%)
Too busy and had no time	63 (43.2%)
Long wait time at clinic or emergency room	48 (32.9%)
Fear of response from leadership	10 (6.8%)
Did your illness have an impact on your performance? Responded “Yes”	154 (53.7%)
*N* Missing	2
If Yes, what was the impact on your performance and for how many days?	
Missed class	
None	73 (47.4%)
1 day	68 (44.2%)
2-3 days	10 (6.5%)
4-7 days	2 (1.3%)
More than 7 days	1 (0.6%)
Decreased study time	
None	36 (23.4%)
1 day	38 (24.7%)
2-3 days	52 (33.8%)
4-7 days	21 (13.6%)
More than 7 days	7 (4.5%)
Missed physical training	
None	50 (32.5%)
1 day	39 (25.3%)
2-3 days	21 (13.6%)
4-7 days	21 (13.6%)
More than 7 days	23 (14.9%)
Decreased leisure activities	
None	33 (21.4%)
1 day	24 (15.6%)
2-3 days	51 (33.1%)
4-7 days	34 (22.1%)
More than 7 days	12 (7.8%)
Did you take medicine for your symptoms?	
Yes, prescribed from healthcare provider	124 (44.8%)
Yes, self-medication	112 (40.4%)
No, no treatment	41 (14.8%)
N Missing	12

The multivariable model indicates that women and younger trainees (<30 years of age) were more likely to report ILI (women: OR 1.58, 95% CI 1.30, 1.92; <30 years old: OR 1.58, 95% CI 1.06, 2.36) (Table III; univariable results in Supplementary Table S2). Trainees in the Army and Navy/Marines were more likely to report an ILI than trainees in the Air Force (OR 1.44, 95% CI 1.09, 1.89; OR 1.60, 95% CI 1.24, 2.06, respectively). In addition, participants who reported longer trainings were more likely to report ILIs, participants who filled out a survey in the summer months were less likely to report ILIs, and participants who received training in 2017/18 and 2018/19 were less likely to report ILIs than participants who received training in 2016/17. A sub-analysis performed only on the individuals who received version 2 of the form identified a protective effect of reported handwashing 10+ times per day (OR 0.61, 95% CI 0.42, 0.89) (Table III). When considering healthcare-seeking behavior for ILIs as the outcome of interest, women were marginally more likely to seek healthcare than men (OR 1.33, 95% CI 0.98, 1.80), although this relationship was not statistically significant (*P* = 0.07). No other factors were related to healthcare-seeking behaviors for ILI.

**TABLE III.** Logistic Regression Model Results with Odds of Reporting Influenza-like Illness (ILI) (Column 1 Includes Everyone Who Received a Survey, Column 2 Includes Only Those Who Received Version 2 of the Survey) or Seeking Healthcare for ILIs (Third Column) during Training as the Outcomes among Military Trainees at Fort Sam Houston. Logistic Regression Models Also Controlled for Month of Survey, Season of Survey (2016/2017, 2017/2018, 2018/2019), and Days of Training (Categorically, <60, 60-89, 90-119, and 120+ Days)

	Outcome = Reported ILI		Outcome = Healthcare sought for ILI
Variable	Full model (*N* = 2,121)	Model using data collected in V2 of survey (*N* = 1,061)	Model among those who reported ILI (*N* = 815)
Intercept	0.62 (0.18, 2.18)	0.29 (0.11, 0.79)*	0.34 (0.06, 1.81)
Age <30 years	1.58 (1.06, 2.36)*	1.10 (0.62, 1.96)	1.24 (0.60, 2.55)
Female	1.58 (1.30, 1.92)^***^	2.03 (1.50, 2.75)^***^	1.33 (0.98, 1.80)
Race/ethnicity: Reference = Asian	–	–	–
Black	0.85 (0.56, 1.29)	1.04 (0.53, 2.03)	0.99 (0.52, 1.89)
Hispanic	1.06 (0.72, 1.58)	0.95 (0.49, 1.84)	0.69 (0.37, 1.27)
Multiple/other race	1.27 (0.80, 2.02)	1.25 (0.60, 2.62)	0.57 (0.28, 1.18)
White	1.39 (0.96, 2.01)	1.13 (0.60, 2.11)	0.80 (0.46, 1.41)
Affiliation: Reference = Air Force	–	–	–
Army	1.44 (1.09, 1.89)*	0.77 (0.50, 1.18)	1.48 (0.92, 2.37)
Navy/Marines	1.60 (1.24, 2.06)^***^	1.34 (0.84, 2.13)	1.27 (0.85, 1.92)
Washes hands 4+ times per day	0.95 (0.70, 1.27)		0.94 (0.60, 1.47)
Washes hands 7-9 times per day		0.78 (0.56, 1.10)	
Washes hands 10 + times per day		0.61 (0.42, 0.89)^**^	
Season: Reference = 2016/2017	–	–	–
2017/2018	0.47 (0.37, 0.58)^***^		1.47 (1.03, 2.11)*
2018/2019	0.29 (0.16, 0.52)^***^	0.91 (0.33, 2.51)	2.27 (0.84, 6.17)
Length of training: Reference=<60 days	–	–	–
60-89 days	1.32 (0.97, 1.79)	2.06 (1.20, 3.55)^**^	0.72 (0.43, 1.18)
90-119 days	1.75 (1.31, 2.34)^***^	2.95 (1.82, 4.77)^***^	1.24 (0.78, 1.97)
120+ days	1.42 (1.04, 1.95)*	1.91 (1.21, 3.01)^**^	1.27 (0.75, 2.16)
Calendar month of survey: return Reference = January	–	–	–
February	0.75 (0.25, 2.22)		0.97 (0.27, 3.46)
March	0.54 (0.18, 1.59)	0.56 (0.27, 1.14)	1.33 (0.36, 4.88)
April	0.38 (0.13, 1.12)	0.65 (0.32, 1.31)	1.04 (0.28, 3.84)
May	0.39 (0.13, 1.18)	0.58 (0.29, 1.19)	1.63 (0.41, 6.49)
June	0.17 (0.05, 0.56)^**^	0.20 (0.08, 0.49)^***^	1.50 (0.33, 6.90)
July	0.19 (0.05, 0.70)*	0.56 (0.15, 2.07)	1.37 (0.24, 7.91)
August	0.25 (0.08, 0.77)*	0.39 (0.19, 0.84)*	1.32 (0.34, 5.07)
September	0.40 (0.12, 1.26)	0.31 (0.10, 1.00)	0.61 (0.14, 2.63)
October	0.59 (0.16, 2.21)	0.73 (0.29, 1.83)	1.53 (0.25, 9.52)

*** *P* < .001; ^**^P < .01; *P < .05.

## DISCUSSION

Healthcare was sought by 40% of the individuals who had ILIs during their training, which is slightly lower than the percentages reported in the literature for the general population (41-47%.)^12–14^ The similarity across studies is consistent with findings from Peppa et al. who reported that the proportion of individuals who seek care for ILIs is relatively stable even as influenza activity and severity varies over time.^15^ This rate of healthcare seeking is, however, slightly higher than that observed in a cohort of similarly aged college students, wherein only 26% with ILI sought healthcare.^16^

ILIs had a negative impact on training according to over half of the participants who reported ILIs. Of trainees who reported an impact on their training, 53% missed at least 1 day of class, and 77% reported a decrease in the amount of time they were able to study as a result of their ILI. This rate of absenteeism is notably lower than in a cohort of similarly aged college students, among whom 58% with ILI missed at least one day of class.^16^ Among those college students with ILI, up to 41% reported doing poorly on a test and up to 58% doing poorly on a class assignment. The lower rate of absenteeism noted in our study may reflect barriers to military trainees removing themselves from classroom activities. Aside from the difference in absenteeism, the level of impairment caused by ILI reported by college students in the earlier study and military trainees herein was similar. Prior studies have shown that upper respiratory infections (URIs) in general decrease effectiveness at work by decreasing subjective alertness and psychomotor functioning.^17,18^ In one study, influenza impaired the ability to perform reaction-time tasks to a degree similar to that associated with alcohol consumption or working at night. In a second study, 88% of workers with URIs reported a mean decrease of 21.2% in work effectiveness.^19^ In a third study, workers with ILI reported a level of work effectiveness of 4.6 on a scale of 1 (low effectiveness) to 10 (normal effectiveness).^20^

The observation that military trainees in this study sought healthcare more often but missed class less often than similarly aged college students with ILI suggests an opportunity to decrease the impact of ILI on this training population. Seeking healthcare early (within 48 hours of symptom onset) can be beneficial in some circumstances because a rapid diagnosis of influenza and provision of antiviral therapy can shorten the duration of symptoms for the individual and decrease the risk of transmission of influenza to others.^21^ During periods of known high influenza activity, proactively identifying trainees with ILI for rapid diagnosis and treatment might therefore be beneficial. In general, policies and processes encouraging those with ILI to self-isolate decrease the spread of viral pathogens, including SARS-CoV-2, within a community. Future studies to elucidate the reasons why trainees attended class despite ILI symptoms are warranted.

More frequent handwashing was associated with a lower risk of reporting ILI only in the sub-analysis of individuals who received version 2 of the survey. Version 1 of the survey asked about handwashing frequency up to >4 times per day, while version 2 categorized the frequency further, including 4-6 times per day, 7-9 times per day, and 10+ times per day. Although no differences were observed between individuals who washed their hands 4+ times per day and those who did not, 10+ times per day appeared to be associated with a decreased risk of reporting ILI in this population. It is likely that better handwashing practices are associated with other hygienic practices that may decrease the risk of ILI acquisition and handwashing has been shown to reduce respiratory infections in both clinical and non-clinical settings.^22–25^ In a handwashing trial implemented at a Navy training center, researchers reported a 45% reduction in respiratory infection-associated clinic visits after the researchers facilitated the distribution of health education messages, encouragement by leadership to wash hands, and increased availability of soap for handwashing.^22^ These handwashing-related interventions, if implemented at a larger scale, have the potential to augment the current influenza and adenovirus vaccination programs and further decrease the burden of respiratory infections in the military.

Certain demographic characteristics were associated with reported ILIs. Women were more likely to report having had an ILI than men, a finding which is consistent with the published literature.^26–30^ The reasons for this difference in risk between the sexes are unclear but may be related to differences in recalling or reporting ILIs. Age was also related to the risk of reporting an ILI. As this was a trainee population, the trainees were mostly young adults, with a median age of 21 years, but some older individuals received training (7% were 30+ years of age). The older trainees were found to have a lower risk of reporting an ILI than the younger trainees, perhaps due to differences in exposure history and unmeasured behavioral factors. Finally, military branch was related to risk of reporting an ILI, with both the Army and Navy/Marines at a higher risk of reporting an ILI than the Air Force. These differences between trainees in different branches persisted even when controlling for age, sex, race, and other characteristics by multivariable analysis. There are likely unmeasured confounders at play in this relationship, perhaps related to exposures outside the classrooms. While the course content, structure, and classroom environment are relatively standardized, other aspects of trainee experience while on Ft. Sam Houston are not. For example, the individual military branches provide living quarters, define physical fitness requirements, and maintain administrative control while the member is in training. The surveys did not assess such variables.

This study has several weaknesses. The surveys were collected at the end of trainings which spanned different lengths of time, without inquiring about the number of ILIs the participant might have experienced during the training, which only allows analysis of a binary outcome. The outcome is dependent on the participant recalling an ILI during the period of the training session, which could be quite long in some cases. In addition, there is no severity or etiology component of the ILI data that were collected; therefore, more detailed analyses cannot be performed. Ideally, one would review medical records to confirm healthcare-seeking behavior and to get more details about the ILI, but that is beyond the scope of this project. However, this is a large survey in a military population that gives us some insights into the burden of respiratory infections in trainees and the consequences of those infections in terms of impaired participation in training.

ILIs appear to be far more common during military training than healthcare records may indicate. Our data suggest increasing access to facilities for hand hygiene and education about the importance of hand hygiene to prevent the spread of disease may reduce the ILI burden in this population and will likely reduce the risk for SARS-CoV-2 transmission. Future work should focus on exploring risk factors identified in this analysis to allow for the development of a comprehensive package of respiratory infection risk reduction techniques in order to decrease the burden of ILIs in this population.

## Supplementary Material

usac016_Supp

## References

[1] GBD 2016 Lower Respiratory Infections Collaborators : Estimates of the global, regional, and national morbidity, mortality, and aetiologies of lower respiratory infections in 195 countries, 1990–2016: a systematic analysis for the Global Burden of Disease Study 2016. Lancet Infect Dis 2018; 18(11): 1191–210. 30243584 10.1016/S1473-3099(18)30310-4 PMC6202443

[2] Gray GC : Acute respiratory disease in the military. Fed Pract 1995; 12(1): 27–33.

[3] Sanchez JL, Cooper MJ, Myers CA, et al: Respiratory infections in the U.S. military: recent experience and control. Clin Microbiol Rev 2015; 28(3): 743–800. 26085551 10.1128/CMR.00039-14 PMC4475643

[4] Gray GC, Callahan JD, Hawksworth AW, Fisher CA, Gaydos JC: Respiratory diseases among U.S. military personnel: countering emerging threats. Emerg Infect Dis 1999; 5(3): 379–85. 10341174 10.3201/eid0503.990308 PMC2640764

[5] O’Shea MK, Wilson D: Respiratory infections in the military. J R Army Med Corps 2013; 159(3): 181–9. 24109140 10.1136/jramc-2013-000110

[6] Yun HC, Young AN, Caballero MY, Lott L, Cropper TL, Murray CK: Changes in clinical presentation and epidemiology of respiratory pathogens associated with acute respiratory illness in military trainees after reintroduction of adenovirus vaccine. Open Forum Infect Dis 2015; 2(3): ofv120. 10.1093/ofid/ofv120 PMC4569648 26380351

[7] Radin JM, Hawksworth AW, Blair PJ, et al: Dramatic decline of respiratory illness among US military recruits after the renewed use of adenovirus vaccines. Clin Infect Dis 2014; 59(7): 962–8. 24991024 10.1093/cid/ciu507

[8] Coleman R, Eick-Cost AA, Hawksworth AW, et al: Department of Defense end-of-season influenza vaccine effectiveness estimates for the 2017-2018 season. Msmr 2018; 25(10): 16–20. 31066571

[9] Flannery B, Chung JR, Belongia EA, et al: Interim estimates of 2017–18 seasonal influenza vaccine effectiveness - United States, February 2018. MMWR Morb Mortal Wkly Rep 2018; 67(6): 180–5. 29447141 10.15585/mmwr.mm6706a2 PMC5815489

[10] Zhang Y, May L, Stoto MA: Evaluating syndromic surveillance systems at institutions of higher education (IHEs): a retrospective analysis of the 2009 H1N1 influenza pandemic at two universities. BMC Public Health 2011; 11: 591. 10.1186/1471-2458-11-591 PMC3151236 21791092

[11] R Core Team : R: a language and environment for statistical computing. 3.6.0 ed. Vienna, Austria: R Foundation for Statistical Computing; 2019. Available at https://www.R-project.org/.

[12] Biggerstaff M, Jhung MA, Reed C, Fry AM, Balluz L, Finelli L: Influenza-like illness, the time to seek healthcare, and influenza antiviral receipt during the 2010-2011 influenza season-United States. J Infect Dis 2014; 210(4): 535–44. 24731959 10.1093/infdis/jiu224 PMC4610008

[13] Ma W, Huo X, Zhou M: The healthcare seeking rate of individuals with influenza like illness: a meta-analysis. Infect Dis (Lond) 2018; 50(10): 728–35. 30009680 10.1080/23744235.2018.1472805

[14] Meng H, Liao Q, Suen LK, O’Donoghue M, Wong CM, Yang L: Healthcare seeking behavior of patients with influenza like illness: comparison of the summer and winter influenza epidemics. BMC Infect Dis 2016; 16: 499. 10.1186/s12879-016-1821-7 PMC5029067 27646778

[15] Peppa M, John Edmunds W, Funk S: Disease severity determines health-seeking behaviour amongst individuals with influenza-like illness in an internet-based cohort. BMC Infect Dis 2017; 17(1): 238. 10.1186/s12879-017-2337-5 PMC5374571 28359335

[16] Nichol KL, D’Heilly S, Ehlinger E: Colds and influenza-like illnesses in university students: impact on health, academic and work performance, and healthcare use. Clin Infect Dis 2005; 40(9): 1263–70. 15825028 10.1086/429237

[17] Smith A, Thomas M, Kent J, Nicholson K: Effects of the common cold on mood and performance. Psychoneuroendocrinology 1998; 23(7): 733–9. 9854744 10.1016/S0306-4530(98)00042-0 PMC7131765

[18] Smith A, Brice C, Leach A, Tiley M, Williamson S: Effects of upper respiratory tract illnesses in a working population. Ergonomics 2004; 47(4): 363–9. 14680996 10.1080/0014013032000157887

[19] Bramley TJ, Lerner D, Sames M: Productivity losses related to the common cold. J Occup Environ Med 2002; 44(9): 822–9. 12227674 10.1097/00043764-200209000-00004

[20] Keech M, Scott AJ, Ryan PJ: The impact of influenza and influenza-like illness on productivity and healthcare resource utilization in a working population. Occup Med (Lond) 1998; 48(2): 85–90. 9614766 10.1093/occmed/48.2.85

[21] Uyeki TM, Bernstein HH, Bradley JS, et al: Clinical practice guidelines by the infectious diseases society of America: 2018 update on diagnosis, treatment, chemoprohylaxis, and institutional outbreak mangaement of seasonal influenza. Clin Infect Dis 2019; 68(6): e1–47. 10.1093/cid/ciy866 PMC6653685 30566567

[22] Ryan MA, Christian RS, Wohlrabe J: Handwashing and respiratory illness among young adults in military training. Am J Prev Med 2001; 21(2): 79–83. 11457626 10.1016/s0749-3797(01)00323-3

[23] Warren-Gash C, Fragaszy E, Hayward AC: Hand hygiene to reduce community transmission of influenza and acute respiratory tract infection: a systematic review. Influenza Other Respir Viruses 2013; 7(5): 738–49. 23043518 10.1111/irv.12015 PMC5781206

[24] Roberts L, Smith W, Jorm L, Patel M, Douglas RM, McGilchrist C: Effect of infection control measures on the frequency of upper respiratory infection in child care: a randomized, controlled trial. Pediatrics 2000; 105(4 Pt 1): 738–42. 10742313 10.1542/peds.105.4.738

[25] Kimel LS : Handwashing education can decrease illness absenteeism. J Sch Nurs 1996; 12(2): 14–6. 10.1177/105984059601200204 8704381

[26] Hardelid P, Rait G, Gilbert R, Petersen I: Recording of influenza-like illness in UK primary care 1995-2013: cohort study. PLoS One 2015; 10(9): e0138659. 10.1371/journal.pone.0138659 PMC4577110 26390295

[27] Wood S, Telu K, Tribble D, et al: Influenza-like illness in travelers to the developing world. Am J Trop Med Hyg 2018; 99(5): 1269–74. 30226131 10.4269/ajtmh.17-0884 PMC6221204

[28] Adler AJ, Eames KT, Funk S, Edmunds WJ: Incidence and risk factors for influenza-like-illness in the UK: online surveillance using Flusurvey. BMC Infect Dis 2014; 14: 232. 10.1186/1471-2334-14-232 PMC4025540 24885043

[29] Ross AM, Kai J, Salter R, Ross J, Fleming DM: Presentation with influenza-like illness in general practice: implications for use of neuraminidase inhibitors. Commun Dis Public Health 2000; 3(4): 256–60. 11280254

[30] Soltis BW, Sanders JW, Putnam SD, Tribble DR, Riddle MS: Self reported incidence and morbidity of acute respiratory illness among deployed U.S. military in Iraq and Afghanistan. PLoS One 2009; 4(7): e6177. 10.1371/journal.pone.0006177 PMC2702682 19584919

