# Supplementary material for: Respiratory Infections Are More Common Than Healthcare Records Indicate: Results From an Anonymous Survey
Source: Mil Med. 2022 Feb 3;188(7-8):e1941–7. doi: 10.1093/milmed/usac016 (PMC9383359; doi:10.1093/milmed/usac016)

**Supplemental table 1. Calendar month of survey collection in respiratory infection study at Fort Sam Houston, by influenza-like illness (ILI) status.**

|  | **Did not report an ILI (N=1306)** | **Reported an ILI (N=815)** | **Total (N=2121)** | **p value** |
| --- | --- | --- | --- | --- |
| **Calendar month of survey** |  |  |  | < 0.01^2^ |
| January | 5 (0.4%) | 14 (1.7%) | 19 (0.9%) |  |
| February | 120 (9.2%) | 153 (18.8%) | 273 (12.9%) |  |
| March | 229 (17.5%) | 210 (25.8%) | 439 (20.7%) |  |
| April | 233 (17.8%) | 153 (18.8%) | 386 (18.2%) |  |
| May | 172 (13.2%) | 90 (11.0%) | 262 (12.4%) |  |
| June | 107 (8.2%) | 28 (3.4%) | 135 (6.4%) |  |
| July | 34 (2.6%) | 12 (1.5%) | 46 (2.2%) |  |
| August | 212 (16.2%) | 87 (10.7%) | 299 (14.1%) |  |
| September | 130 (10.0%) | 41 (5.0%) | 171 (8.1%) |  |
| October | 64 (4.9%) | 27 (3.3%) | 91 (4.3%) |  |

**Supplemental Table 2. Univariable logistic regression model results with odds of reporting ILI (column 1 includes everyone who received a survey, column 2 includes only those who received version 2 of the survey) or seeking healthcare for ILIs (third column) during training as the outcomes among military trainees at Fort Sam Houston. Univariable logistic regression models also controlled for month of survey, season of survey (2016/17, 2017/18, 2018/19), and days of training (categorically, <60, 60-89, 90-119, and 120+ days) (results not shown).**

|  | Outcome=Reported ILI | | Outcome=Healthcare sought for ILI |
| --- | --- | --- | --- |
| Variable | Model using all data (N-2121) | Model using data collected in V2 of survey (N=1061) | Model among those who reported ILI (N=815) |
| Age <30 years | 1.77 (1.19, 2.63)^**^ | 1.32 (0.76, 2.31) | 1.23 (0.61, 2.48) |
| Female | 1.46 (1.21, 1.76)^***^ | 1.87 (1.40, 2.50)^***^ | 1.29 (0.96, 1.72) |
| Race/ethnicity: Reference=Asian |  |  |  |
| Black | 0.92 (0.61, 1.38) | 1.17 (0.61, 2.24) | 0.98 (0.52, 1.86) |
| Hispanic | 1.15 (0.78, 1.70) | 1.08 (0.57, 2.05) | 0.68 (0.37, 1.26) |
| Multiple/Other race | 1.30 (0.83, 2.05) | 1.38 (0.67, 2.85) | 0.55 (0.27, 1.13) |
| White | 1.44 (1.00, 2.07)^*^ | 1.30 (0.70, 2.39) | 0.78 (0.45, 1.37) |
| Affiliation: Reference=Air Force |  |  |  |
| Army | 1.32 (1.01, 1.73)^*^ | 0.69 (0.45, 1.04) | 1.42 (0.90, 2.25) |
| Navy/Marines | 1.51 (1.18, 1.94)^**^ | 1.18 (0.75, 1.85) | 1.23 (0.83, 1.84) |
| Washes hands 4+ times per day | 0.99 (0.74, 1.32) |  | 0.99 (0.64, 1.54) |
| Washes hands 7+ times per day |  | 0.84 (0.60, 1.17) |  |
| Washes hands 10+times per day |  | 0.73 (0.51, 1.05) |  |

Supplemental Figure 1. Version 1 of the survey used in this study


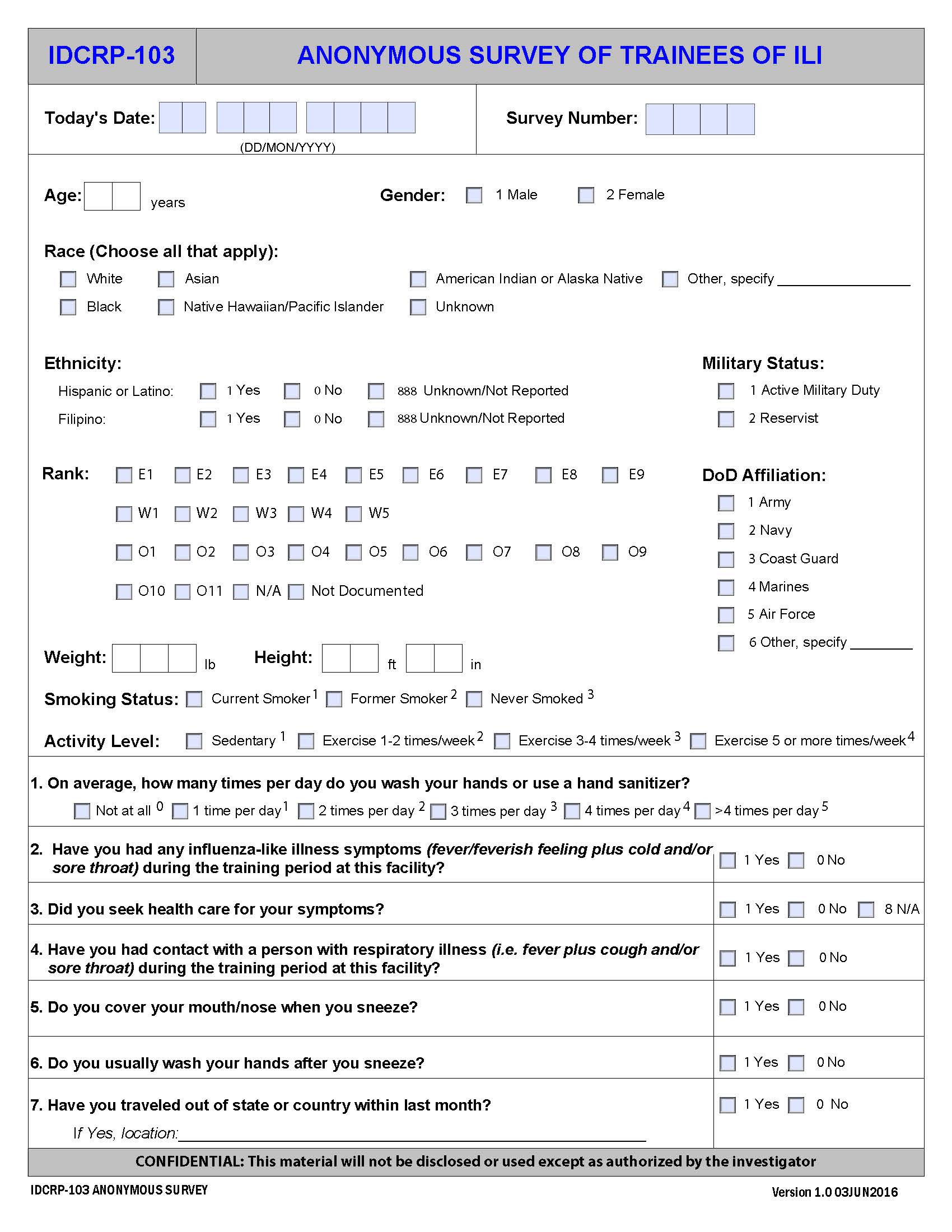


Supplemental Figure 2. Version 2 of the survey used in this study


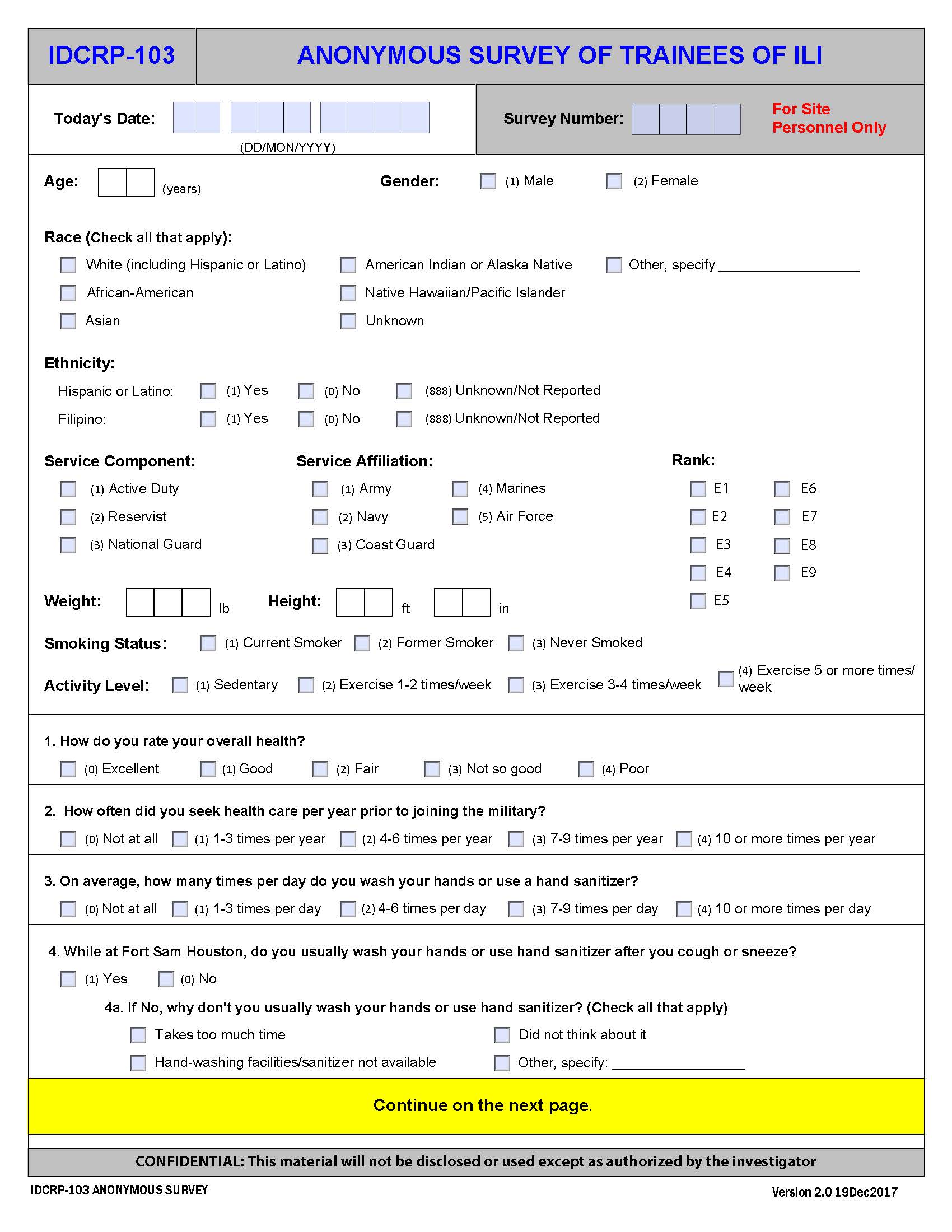


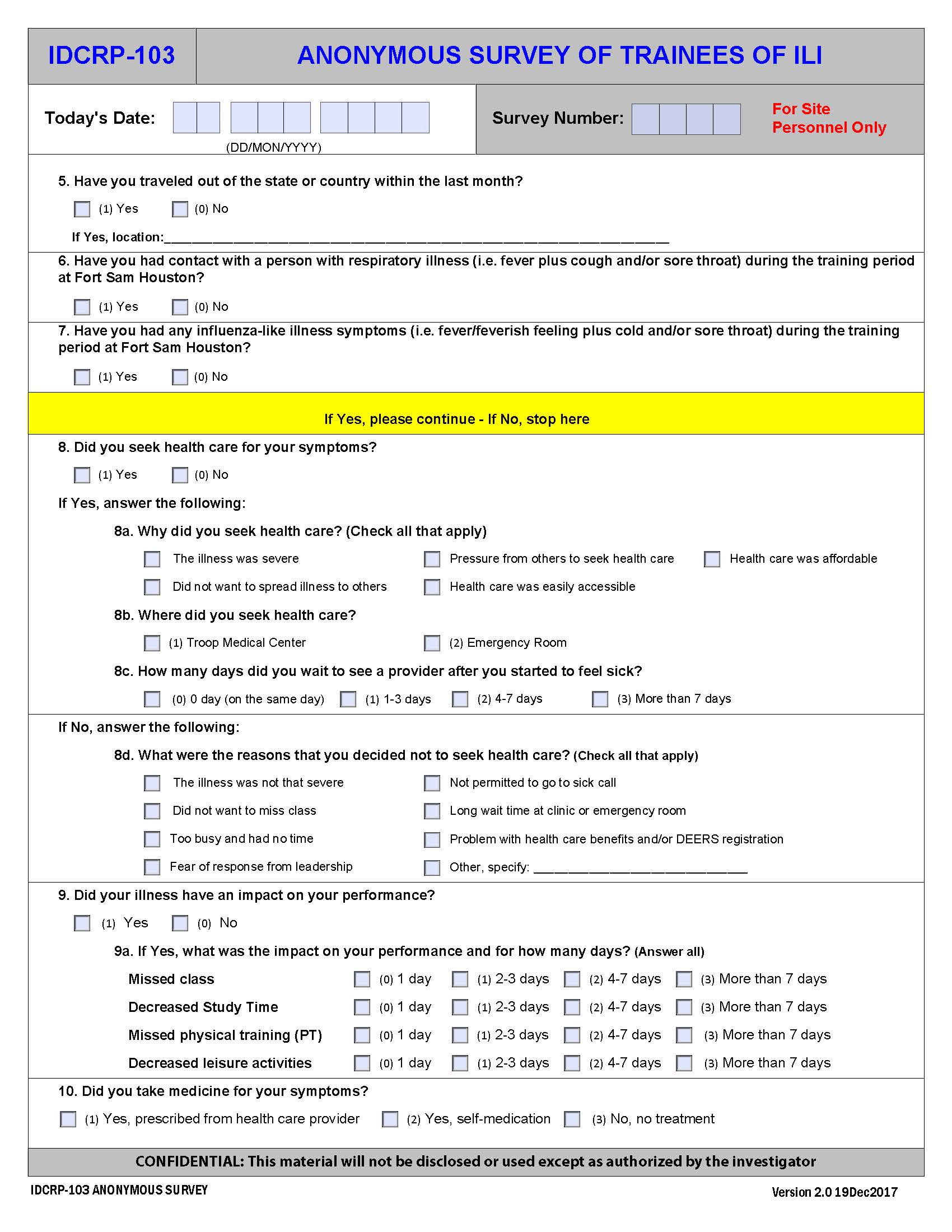

Supplement: usac016_Supp [file usac016_supp.zip › Supp Tables 20211222.docx]
